# Engineering 3D adipose cell models by support bath bioprinting of mature adipocytes in gellan gum hydrogels

**DOI:** 10.48130/els-0026-0006

**Published:** 2026-08-10

**Authors:** Sophia Nowakowski, Franziska B. Albrecht, Petra J. Kluger

**Affiliations:** 1 Institute of Interfacial Process Engineering and Plasma Technology (IGVP), University of Stuttgart, Stuttgart, Germany; 2 Faculty of Natural Science, University of Hohenheim, Stuttgart, Germany; 3 Reutlingen Research Institute, Reutlingen University, Reutlingen, Germany; 4 Fraunhofer Institute for Interfacial Engineering and Biotechnology (IGB), Stuttgart, Germany; 5 Faculty of Life Sciences, Reutlingen University, Reutlingen, Germany

**Keywords:** Adipose tissue engineering, Agarose support bath, Biofabrication, Extrusion, Gellan gum bioink

## Abstract

Mature adipocytes (ACs) are essential for modeling human adipose tissue physiology *in vitro*, yet their large size, buoyancy, and shear sensitivity make them challenging to incorporate into engineered three-dimensional (3D) constructs. This study introduces an agarose support-bath (SB) extrusion workflow for bioprinting geometrically defined adipose constructs using a gellan gum (GG) bioink. We examined the viscosity of GG solutions and SBs to identify a low-shear printability window suitable for lipid-laden cells. SB printing significantly enhanced filament stability, pore uniformity, and construct accuracy compared to free extrusion. Viability assays showed that ACs remained intact and metabolically active after 3 d post-printing, with minimal membrane damage and maintained unilocular morphology. Immunofluorescence staining revealed uniform lipid-filled cells and expression of perilipin A, while basal and stimulated glycerol release indicated retained metabolic activity. These findings demonstrate that the established SB extrusion protocol enables gentle, precise deposition of ACs using a GG-based bioink. This workflow offers a reproducible and accessible platform for generating physiologically relevant adipose cell models, supporting early-stage biological assessment, preliminary drug testing, and soft tissue engineering.

## Introduction

Adipose tissue is a dynamic organ with critical roles in energy homeostasis, endocrine signaling, and metabolic regulation. It is composed of various cell types, including ACs, adipose-derived stem cells (ASCs), immune cells, and endothelial cells, which form lobular structures^[1]^. ACs constitute the largest cell population in adipose tissue and influence systemic metabolism, while their dysfunction contributes to multiple diseases, like obesity, insulin resistance, and type 2 diabetes^[2]^. Consequently, there is a strong biomedical interest in developing human-relevant *in vitro* models that capture cellular organization and metabolic behavior to study disease mechanisms and test drugs^[3]^. However, realizing such models requires advanced biofabrication tools capable of spatially organizing fragile, buoyant cells in three dimensions while preserving their viability, morphology, and function.

Despite significant progress in adipose tissue engineering, reproducing the structural and mechanical features of native adipose tissue remains a challenge. Conventional two-dimensional (2D) cultures fail to reproduce the native three-dimensional (3D) architecture, soft mechanical environment, and buoyancy effects of lipid-filled cells^[4]^. Animal models provide a physiological context but are species-specific and entail both ethical and financial burdens^[5]^. Most 3D adipose tissue constructs rely on stem cells, as they can be proliferated and adipogenically differentiated, and are less mechanically sensitive than ACs^[6]^. Using ACs for adipose tissue engineering is favorable because they are readily available after isolation and are fully differentiated and functional^[7]^. The use of ACs poses challenges, as they are relatively large, buoyant, hydrophobic, and shear-sensitive, making them difficult to integrate into engineered 3D constructs and underscoring the need to modify conventional bioprinting methods.

Extrusion-based bioprinting provides spatial control and reproducibility for the fabrication of 3D tissues^[8]^. A specialized variant, support-bath (SB) bioprinting, deposits cell-laden bioinks within a yield-stress, self-healing suspension matrix, allowing for soft or buoyant bioinks (which would collapse under gravity) to maintain structure^[8]^. Different SB systems have been explored for soft tissues, such as neural or cardiac analogues^[9]^, but their application to ACs remains largely unexplored. In a study by Louis et al., ACs, along with vascular cells, were bioprinted in a gellan gum-based SB, demonstrating viability and fatty acid uptake^[10]^. Another study by Albrecht et al. explored the use of methacrylated gelatin as a bioink for printing ASCs and ACs without SB^[11]^. Several approaches for printing adipose tissue models have been applied, differing mainly in the cells used and the materials selected (Table 1). In terms of materials, different polymeric hydrogel materials, such as alginate, gelatin, or collagen type I, are used as bioinks in adipose tissue engineering, depending on the cell type and construct geometry^[12]^. Further, decellularized extracellular matrix offers a cell-specific environment more similar to the native state^[13]^. Among them, gellan gum (GG) is a promising bioink candidate due to its natural polysaccharide properties, including tunable gelation, shear-thinning behavior, and biocompatibility^[8]^. A study by Albrecht et al. shows that GG supports the differentiation of adipose-derived stem cells into unilocular adipocytes in long-term culture^[14]^. Recent work of a defined inflamed adipose cell model shows that AC stability decreases with increasing GG concentration, highlighting the use of low GG concentrations (< 0.4% w/v) for soft tissues^[15]^. Despite these advances, almost no standardized workflow currently exists for bioprinting ACs with defined construct geometry, reproducible fidelity, and accessible readouts. Most studies focus on stem cells, leaving a gap in material-driven, scalable methods for AC-based constructs.

**Table 1:** State-of-the-art bioprinting of adipose tissue.

Model	Printing technique	SB	SB material	Bioink composition	Cross-linking	Cells used	Culture period (d)	Research highlights	Application	Ref.
ACs										
*In vitro* adipose cell model	Extrusion	×	n.a.	GelMA	UV-light (LAP)	ACs ASCs	8 15	Bioprinting process had no negative effects on differentiation potential or lipid maintenance Use of ACs resembles the native adipose tissue state more	More physiological *in vitro* adipose cell models	[11]
Vascularized AT model	Extrusion	✓	Gellan gum	Fibrinogen collagen microfibers	Enzymatic (thrombin)	ACs ASCs HUVECs	7	First documented bioprinting of ACs Maintenance of AC viability and lipid functionality Generation of early vascular networks by co-printing with ECs	Soft tissue regeneration (clinical application)	[10]
Microtissues										
Autologous fat graft	Extrusion	×	n.a.	Fibrinogen gelatin HA PCL	Enzymatic (thrombin)	Adipose micro-fragments	14 *in vitro*; 28 *in vivo*	Development of a tissue micronizer for defined fragments Creation of a viable microfragment-based bioink Printing of a patient-specific AT graft and in vivo testing	Patient-specific volumetric soft tissue reconstruction (clinical application)	[23]
Fat graft	Extrusion	×	n.a.	Alginate nanocellulose	Ionic (CaCl_2_)	Lipoaspirate-derived AT	30	Printable lipoaspirate-based bioink containing intact ASCs and ACs Neovascularization of the implanted graft	Soft tissue regeneration (clinical application)	[37]
ASCs										
Life-like AT constructs	Stereolithography	×	n.a.	GelMA HA	UV-light (LAP)	ASCs Fibroblasts HUVECs	27	Multi-material printing platform 3D adipose tissue models with lumen	Breast reconstruction (clinical application)	[38]
Vascularized AT graft	Extrusion	✓	Alginate	HA Pluronic F-127 gelatin PCL	Ionic (CaCl_2_)	ASCs	56 *in vivo*	ASC spheroids significantly enhance secretion of angiogenic factors *in vivo*	Soft tissue reconstruction (clinical application)	[39]
Vascularized adipose cell model	Extrusion FRESH	✓	Alginate	Collagen gelatin alginate PLLA-PLGA	UV-light (LAP) Ionic (CaCl_2_)	ASCs HAMECs	7	Fabrication of thick 3D adipose tissue constructs with perfusable vascular trees	Complex, multi-cellular tissue model engineering (clinical application)	[40]
*In vitro* densely packed AT model	Extrusion	✓	Alginate	dECM	Ionic (CaCl_2_)	ASCs Monocytes	28	Highly matured adipose constructs Recapitulation of obesity-associated pathological changes *in vitro* (immune component)	Obesity research	[41]

**Figure 1:**
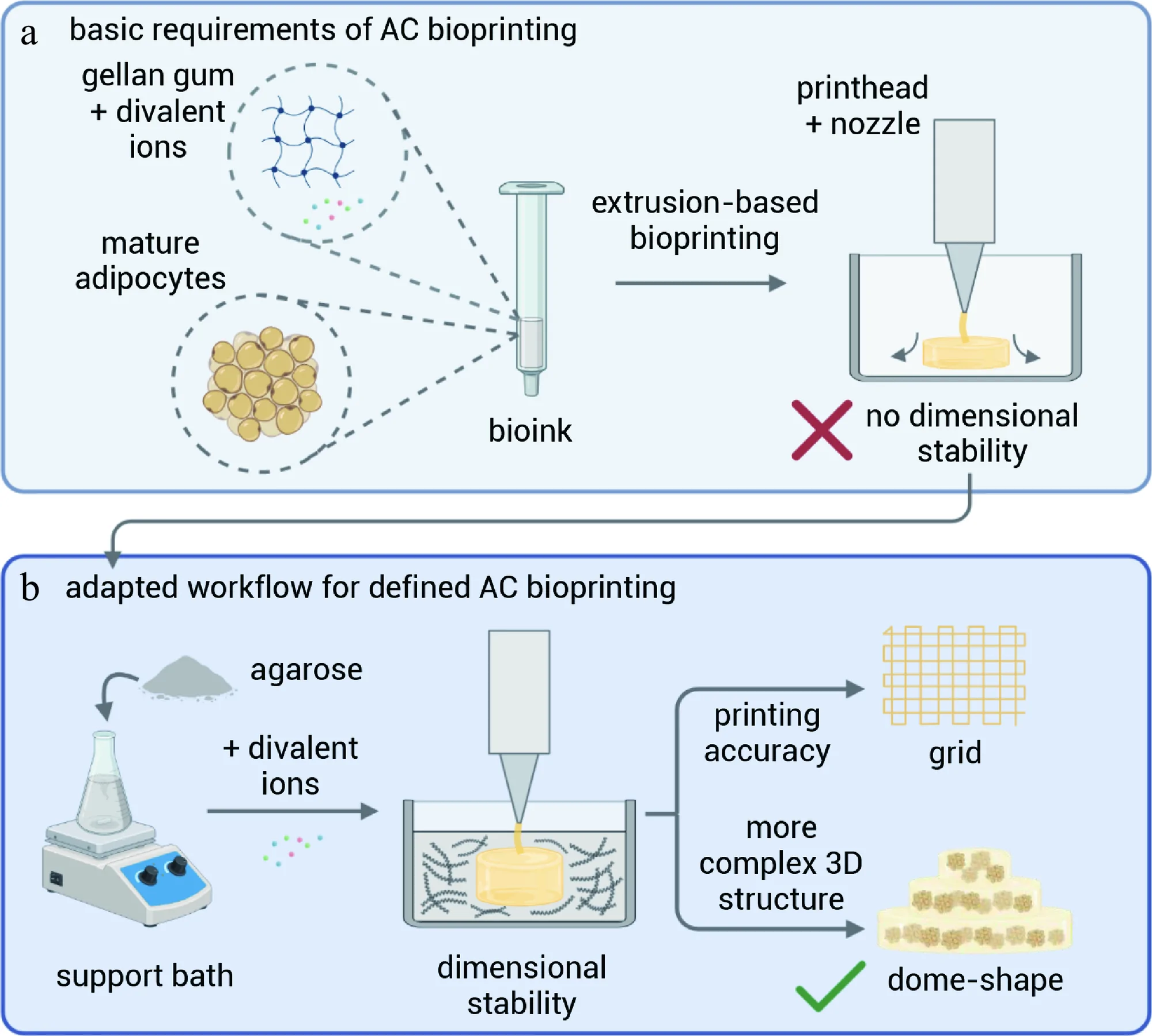
Schematic overview of the biofabrication strategy for mature adipocyte (AC) bioprinting. (a) Conventional extrusion-based bioprinting of gellan gum-based bioinks containing mature adipocytes lacks dimensional stability due to the softness and buoyancy of the bioink. (b) An adapted workflow employing a yield-stress agarose-based support bath with divalent ions enables stabilized extrusion, resulting in improved printing accuracy demonstrated by the fabrication of defined grid structures and more complex three-dimensional dome-shaped constructs.

This study aims to develop and characterize an accessible SB extrusion workflow for printing ACs using a GG-based bioink (Fig. 1). Specifically, we characterized the viscosity of GG bioinks and SB, optimized bioprinting parameters, evaluated construct fidelity with and without SB, and assessed AC viability, morphology, and metabolic function post-printing. We hypothesize that SB extrusion with rheologically compatible GG bioinks allows low-shear deposition of ACs, maintaining their viability, morphology, and metabolism, and thus offers a reproducible protocol for 3D adipose tissue biofabrication.

## Materials and methods

### Isolation and culture of mature adipocytes

Human adipose tissue was provided by Dr. Ziegler and team (Robert Bosch Krankenhaus, Stuttgart, Germany). All research was conducted in accordance with the Declaration of Helsinki on human medical research. Patients provided written consent after being informed about the use of their samples. This is under the permission of the Landesärztekammer Baden-Württemberg (F-2012-078; for normal skin from elective surgeries). ACs were isolated according to a previously described protocol^[16]^. Briefly, fat lobules were dissected from the adipose tissue sample, cut into small pieces, and digested with a collagenase I NB4 (Nordmark Biochemicals) solution (1% bovine serum albumin [BSA, fraction V, Serva Electrophoresis GmbH], 100 Units collagenase in Dulbecco's modified Eagle medium [DMEM, PAN Biotech]) for 1.5 h at 37 °C with light agitation. The digested tissue was sieved, and several washes with DMEM without phenol red were performed. The freshly isolated ACs were allowed to settle for 1 h in DMEM/F12 with 10% FCS and 1% P/S to inactivate any residual collagenase activity, washed with PBS^+^ (Capricorn Scientific), and then immediately used for further experiments. Demographics for all tissue donors are provided in Supplementary Table S1.

### Bioink and support bath formulation

For the GG-based bioink, GG (Gelzan^TM^ CM, Sigma-Aldrich) was dissolved in double-distilled water and 20% PBS^+^ to 0.54% w/v by boiling until the solution was clear. The GG solution was tempered at 40 °C and homogeneously mixed with the AC solution at a 5:4 ratio by pipetting up and down (final GG concentration of 0.3%). The exact amount for one cartridge was 1,725 µL GG mixed with 1,380 µL AC solution. The AC-bioink was transferred into a 3 mL bioprinter cartridge (Nordson) using a 3 mL Luer syringe (Fisher Scientific) via an adapter piece. After attaching a 20 G-nozzle (diameter 0.6 mm, GONANO Dosiertechnik) to the cartridge, it was inserted into the printhead. The support bath consists of 0.5% w/v agarose (Fisher Scientific) in 100% PBS^+^ (adapted from Chrenek et al.^[17]^). The agarose was weighed into Schott flasks, PBS^+^ was added, and the solution was autoclaved at 120 °C for 1.5 h. The still-warm solution was stirred overnight at 700 rpm on a magnetic stirrer. After stirring, the agarose support bath was stored at 4 °C until further use.

### Rheological measurements

The dynamic viscosity of the support bath and acellular gellan gum solutions (0.3% w/v) was measured with a MCR 301 Rheometer (Anton Paar) using a Cone-Plate measuring geometry with 60 mm diameter and 1° cone angle (CP60-1 and P-PTD200). After adding the samples into the measurement gap (0.199 mm), they were left for 3 min to relax and adjust to the set temperature. Support bath solutions were measured at 10 °C, and gellan gum solutions were measured at 20 °C. Viscosity was measured at a shear rate ramp from 0.1 to 100 s^−1^ on a logarithmic scale.

### Bioprinting parameters and design

For extrusion-based bioprinting, the BioX printer (Cellink) was used. The print templates were created as computer-aided design (CAD) files using Autodesk Tinkercad 2025. For printing accuracy measurements, a 20 mm × 20 mm × 1 mm grid template (Supplementary Fig. S1a) with a 15% infill density was printed. For cell culture experiments, a 10 mm × 10 mm × 6 mm dome-shaped octagon (Supplementary Fig. S1b) with a 50% infill density was produced. The printer was calibrated using BioX's automatic calibration program with a plastic cell culture dish (100 mm diameter) as the print vessel. The support bath (about 60 mL) was poured into the Petri dish, and the print bed was cooled to 10 °C. The cartridge filled with bioink was loaded into the printhead, and constructs were printed at a pressure of 4–6 kPa with a speed of 14–16 mm/s. Printed constructs were incubated for at least 30 min in the agarose bath at 37 °C and 5% CO_2_. Then, 1 mL of 50 mM CaCl_2_ solution was carefully pipetted around each construct and incubated for 10 min at 37 °C. After incubation, the constructs were recovered from the support bath with a large spatula and washed twice in PBS^+^. The printed and recovered AC constructs were cultured in a defined medium (endothelial cell basal medium, PB-BH-100-9806 + defined supplement mix, PB-C-SH-042-9799-M, PELOBiotech) for up to 8 d at 37 °C and 5% CO_2_.

### Printing accuracy and image quantification

For printing accuracy measurements, a 20 mm × 20 mm × 1 mm grid was printed and photographed. The dimensions of the printed grid, construct length, pore width, and filament width (three images per biological donor) were analyzed using ImageJ and compared with the design template. The deviation from the template values was calculated using the formula:

$ Deviation_i\left(\%\right)=\dfrac{Measured_i-Design}{Design}\times100 $

### Live/dead staining

For live/dead staining of printed AC constructs, the staining solution was prepared following the manufacturer's instructions (LIVE/DEAD^TM^ Viability/Cytotoxicity Kit, Invitrogen): 1 mL PBS^+^ was supplemented with 0.5 µL calcein, 2 µL ethidium homodimer, and 1 µL Hoechst 33342 and incubated for 1 h at room temperature (RT) in the dark. Microscopic analysis was performed using an Axio Observer microscope and an Axiocam 305 color camera, with ZENblue software (Carl Zeiss).

### Staining of intracellular lipids

Intracellular lipids were stained with BODIPY 493/503 dye (Cayman Chemicals). Tissue samples were fixed with 4% paraformaldehyde (Roti®Histofix, Carl Roth) for 3 h and washed with PBS^+^. Fixed gels were incubated with staining solution (1 mL PBS^+^ supplemented with 1 µL BODIPY and 1 µL Hoechst) for 1 h at RT in the dark, followed by two 15-min washes with PBS^+^. Stained samples were stored at 4 °C until microscopic examination.

### Antibody staining of perilipin A

For anti-perilipin A staining, fixed GG-AC gels were permeabilized for 30 min at RT in a permeabilization solution containing 0.1% Triton-X (Sigma-Aldrich) in PBS^+^. The permeabilized gels were then blocked with a blocking solution of 3% bovine serum albumin and 0.1% Triton X-100 in PBS^+^ for 30 min, followed by three 10-min washes with washing buffer containing 0.1% Tween in PBS^+^. The primary antibody, rabbit anti-human perilipin A (Sigma-Aldrich, P1998), was diluted 1:500 in blocking solution and incubated overnight at 4 °C. After three washing steps, the secondary antibody, goat anti-rabbit IgG-Alexa488 (Abcam, ab60314), was diluted 1:500 in blocking solution and incubated for 30 min at RT in the dark. The gels were then washed and counterstained with 4',6-diamidino-2-phenylindole (DAPI, 1:1,000, Serva Electrophoresis GmbH) for nuclei visualization. Following three washes, the gels were stored in PBS^+^ at 4 °C until microscopic examination.

### Image quantification

Five images (live/dead staining) from each biological donor were manually counted and displayed graphically for semi-quantification of viability. The percentage of viability was determined by counting the nuclei and live cells and calculating their ratio.

### Lactate dehydrogenase assay

LDH release in cell culture supernatant was determined using an LDH assay kit (Cytotoxicity Detection Kit [LDH], Roche). Cell culture supernatant and LDH reagent (1:50 catalyst: dye) were mixed 1:1 in a 96-well plate. Samples treated with 1% Triton X-100 were used as controls. The plate was incubated in the dark for 30 min, then measured at 480 nm, with a 680 nm wavelength correction applied using the SpectraMax i3 plate reader (Molecular Devices).

### Glycerol assay

Glycerol release was quantified using an assay kit (Randox Laboratories) according to the manufacturer's instructions. The cell culture supernatants (unstimulated and stimulated with 10 µM isoproterenol (isoproterenol hydrochloride, Fisher Scientific) for 24 h) were diluted in buffer (1:5), and a standard series in buffer (0–100 ng/mL) was prepared. Diluted samples were mixed with glycerol assay reagent (1:1) and incubated in the dark for 10 min. Samples were measured in triplicate in a 96-well plate at 520 nm.

### Statistics

Data were obtained from five independent experiments with five different biological donors. All values are shown as mean ± standard deviation. Data from the LDH assay were analyzed for significance using an unpaired t-test with Welch's correction. Data from the glycerol assay were analyzed for significance using a two-way analysis of variance (ANOVA) followed by a post hoc test (Šidák). Statistics were conducted with GraphPad Prism 10.5.0. Results were stated as statistically significant when * *p* ≤ 0.05, ** *p* ≤ 0.01, or *** *p* ≤ 0.001.

## Results

### Viscosity mapping of bioinks and support baths defines an adipocyte-friendly printability window

**Figure 2:**
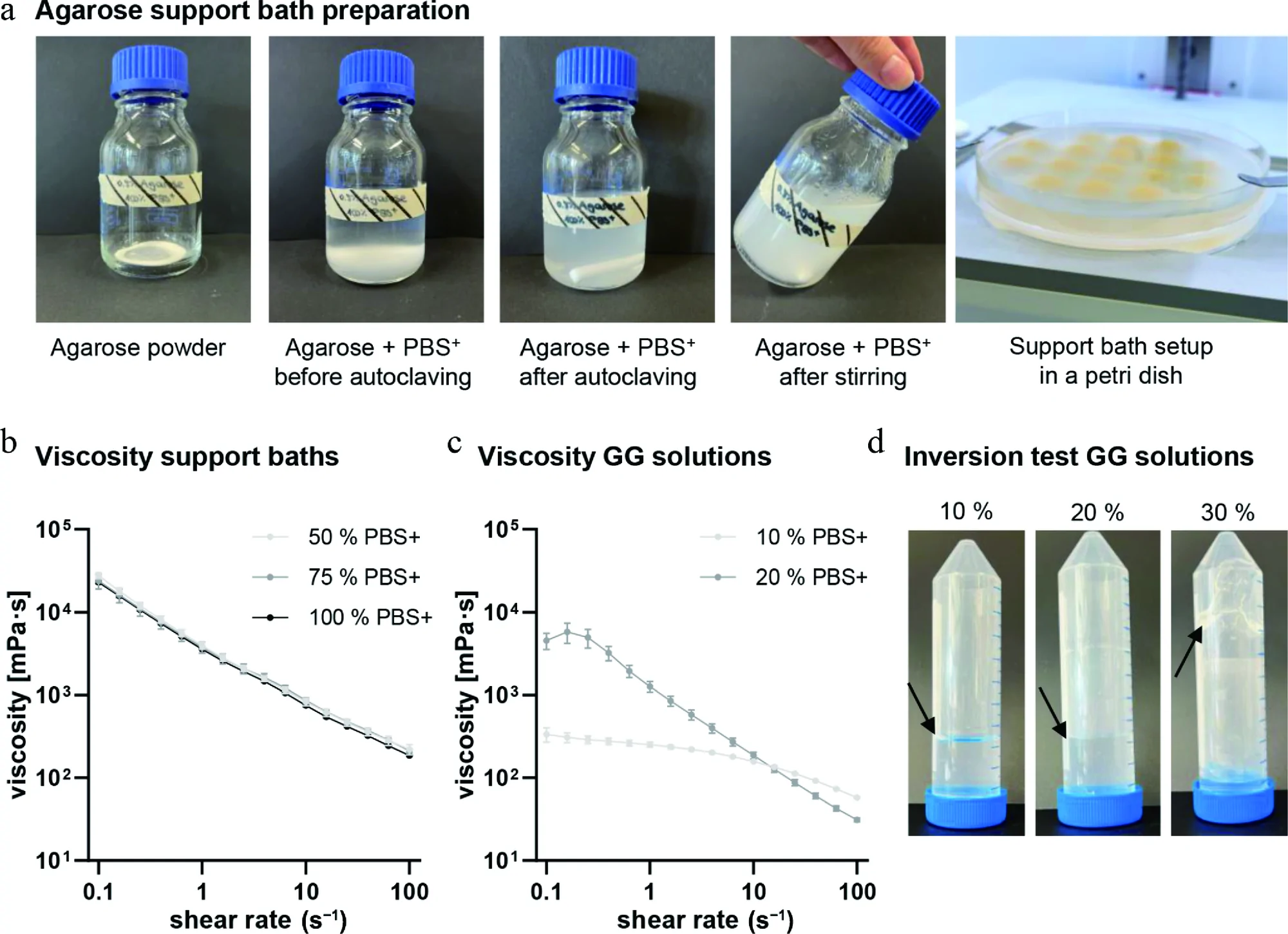
Support bath preparation and rheological characteristics of agarose support baths and gellan-based bioinks for printing mature adipocytes. (a) Preparation of agarose support bath by mixing agarose with PBS^+^, autoclaving it, and stirring it overnight. (b) Viscosity of different support bath compositions (50%, 75%, and 100% PBS^+^) at 10 °C. (c) Viscosity of different acellular GG solutions (10% and 20% PBS+ in stock solution) at 20 °C. (d) Inversion test of different acellular GG solutions (10%, 20%, and 30% PBS+ in stock solution) at room temperature (20 °C). Black arrows indicate the GG solution level after the Falcon tubes were inverted. Each analysis was performed in triplicate. Mean values represent the mean ± standard deviation.

To define the printability window for support-bath extrusion of mature adipocytes, the rheological properties of acellular GG-based bioinks and support-bath formulations with varying initial PBS^+^ concentrations were characterized. Understanding viscosity and yield stress properties is essential to ensure low-shear deposition while maintaining the construct shape during embedded printing. Therefore, rotational rheology measurements were performed to determine whether the materials can be extruded under low shear, which is suitable for printing sensitive ACs. Agarose was chosen as the support bath material based on a study by Chrenek et al., with slight modifications to the composition and manufacturing protocol (Fig. 2a). Agarose (0.5% w/v) was mixed with different ratios of PBS^+^ (50%, 75%, 100%) and double-distilled water, showing no mixing at room temperature. After autoclaving, the agarose solution was turbid and thickened with stirring overnight. The viscosity of the agarose support baths decreased with increasing shear rate, indicating shear-thinning behavior, as required for printing delicate cells (Fig. 2b). There was no significant difference in viscosity between the different SB compositions. The bioinks tested in this study are gellan gum-based (final concentration of 0.3% w/v) with different ratios of PBS^+^ in the stock solution (10%, 20%, 30%). The viscosity of the acellular GG solution with 10% PBS^+^ was lower than that with 20%, and the solution with 30% already gelled at the measurement temperature of 20 °C, so it could not be measured (Fig. 2c). This behavior was also observed in the inversion test, where GG solutions with 10% and 20% PBS^+^ remained liquid at 20 °C, and with 30% PBS^+^, a gel formed that resisted gravity (Fig. 2d). The rheological measurements did not reveal any difference in the viscosity of the tested support baths, so the composition with the highest PBS^+^ concentration was chosen to enhance post-crosslinking of the cell-containing constructs.

### Support-bath printing enhances geometric fidelity and adipocyte retention compared to free extrusion

**Figure 3:**
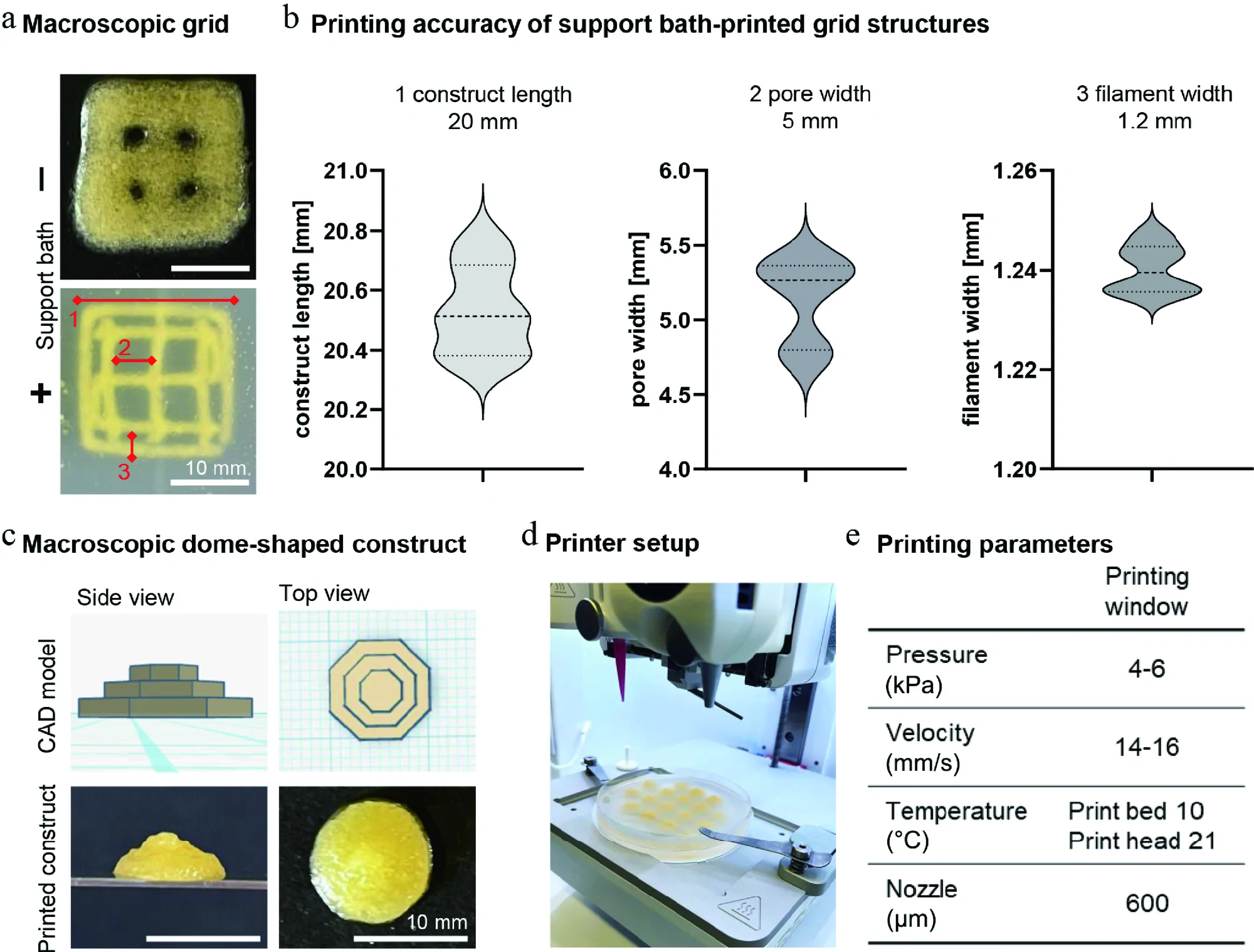
Bioprinting characteristics and printing protocol. (a) Macroscopic images of grid structures printed without (−) and with (+) support bath; red markings and numbers highlight the analyzed parameters for printing fidelity. Scale bar: 10 mm. (b) Analysis of (1) construct length, (2) pore width, and (3) filament width of support-bath printed grid structures. Design parameters for each measure are indicated above each graph, with a construct length of 20 mm, a pore width of 5 mm, and a filament width of 1.2 mm. (c) Macroscopic pictures of dome-shaped constructs in side and top view with CAD model and support-bath printed and recovered construct. Scale bar: 10 mm. (d) Overview of the printer setup using the BioX printer and a Petri dish as printing vessel. (e) Listed bioprinting windows for printing AC constructs. Mean values represent the mean ± SD of six independent experiments for accuracy measurements of printed grid structures.

Based on the rheological insights, we next evaluated how the acellular material properties translated into practical printability by examining filament formation, grid fidelity, and overall construct geometry of the cell-laden bioink after SB extrusion. This was done with the selected SB composition containing 100% PBS^+^ and the GG-based bioink with 20% PBS^+^ in the stock solution. The SB-based printing protocol for ACs enables the printing of defined grid structures (20 mm × 20 mm × 1 mm, Supplementary Fig. S1a) compared to free extrusion, where the cell-laden ink spreads and layering is not possible (Fig. 3a). To quantify the printing accuracy of the SB-printed grid structures, the actual construct length, pore width, and filament width were compared to the target values (Fig. 3b). The actual construct length deviated by 2.66% ± 1.12% from the designed length of 20 mm (20.32 ± 0.23 mm). The measured pore width differed by 2.87% from the target value of 5 mm, with an actual overall value of 5.14 ± 0.30 mm. The filament width deviated by 3.35% ± 1.49% from the target value of 1.2 mm (1.24 ± 0.02 mm). For the following biological evaluations, a more complex 3D construct was designed as a dome-shaped octagon (10 mm × 10 mm × 6 mm; Supplementary Fig. S1b). The SB-printed and recovered dome-shaped constructs showed a comparable appearance to the designed CAD model (Fig. 3c). The BioX printer setup, along with a Petri dish containing the SB, enables higher throughput of constructs in a single run (Fig. 3d). The printing parameters were narrowed to a small window for applied pressure and printing velocity, with a cold SB for additional gelation support and a nozzle diameter of 600 µm (Fig. 3e). This SB-based extrusion workflow enabled reproducible biofabrication of AC-laden constructs with balanced printing precision, which were subsequently analyzed for biological performance.

### Printed adipocyte constructs show preserved viability with minimal cytotoxicity post-printing

**Figure 4:**
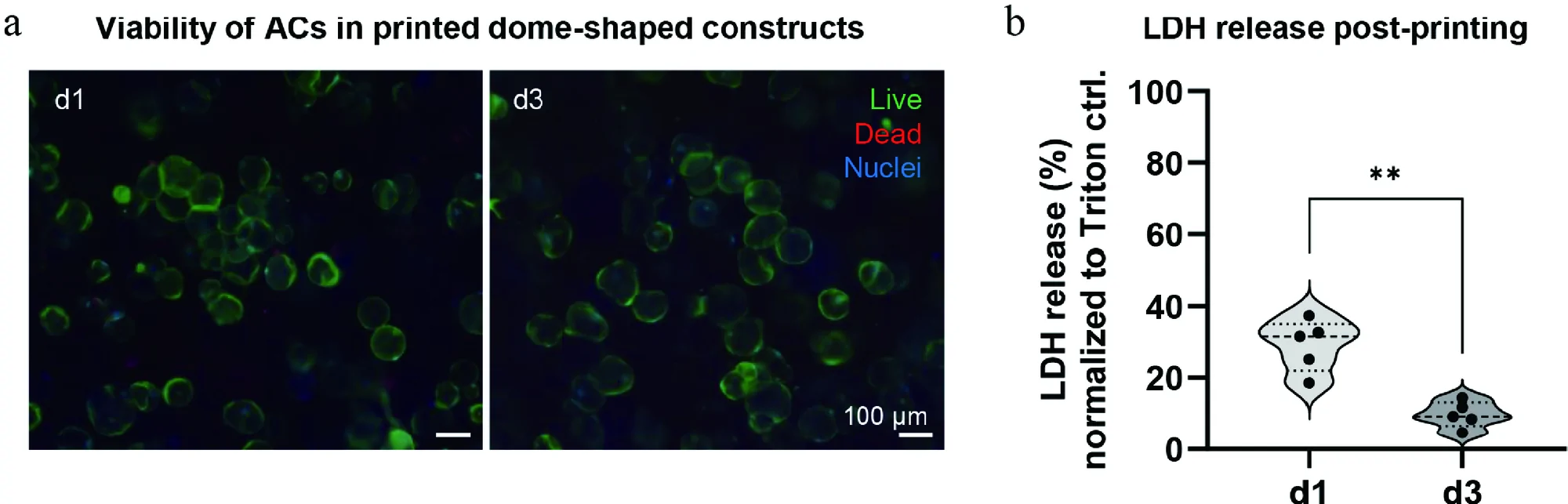
Viability of mature adipocytes in the printed dome-shaped constructs. (a) Live/dead staining of printed dome-shaped constructs on days 1 and 3 post-printing, living cells in green, dead cells in red, and cell nuclei in blue. Scale bar: 100 µm. (b) Normalized LDH release of printed dome-shaped constructs on days 1 and 3 post-printing, normalized to 1% Triton X-100 control (set to 100%). Data are represented as the mean ± standard deviation, with single values shown as dots from five independent experiments with five different biological donors. ** *p* ≤ 0.01.

The printed dome-shaped constructs were recovered from the SB using several simple wash steps with PBS^+^ and cultured for up to 3 d in a defined medium established in a previous study^[15]^. As ACs are a mechanically sensitive and buoyant cell type, the suitability of the printing process was evaluated by analyzing the viability of the AC constructs at days 1 and 3 post-printing (Fig. 4). Live/dead staining of encapsulated ACs showed a high proportion of viable ACs for 3 d, with very few dead cells visible (Fig. 4a; Supplementary Fig. S2a, S2b). Semiquantitative viability evaluation confirmed sustained viability until day 3 (Supplementary Fig. S2c). As a snapshot of a potential long-term culture, viable cells were observed up to day 8 of culture (Supplementary Fig. S3). During cell turnover, elevated levels of free lactate dehydrogenase (LDH) can correlate with compromised cell membranes, indicating cell stress or death. On day 1 post-printing, normalized LDH release was significantly elevated (29.09% ± 9.03%) compared to day 3. LDH levels on day 3 decreased to 9.66% ± 4.54%, indicating cell recovery (Fig. 4b), as confirmed by live/dead staining. These findings qualify the printing process for delicate ACs, with high viability after printing and SB recovery.

### Printed adipocyte constructs exhibit unilocular morphology and maintain metabolic activity over 3 days

**Figure 5:**
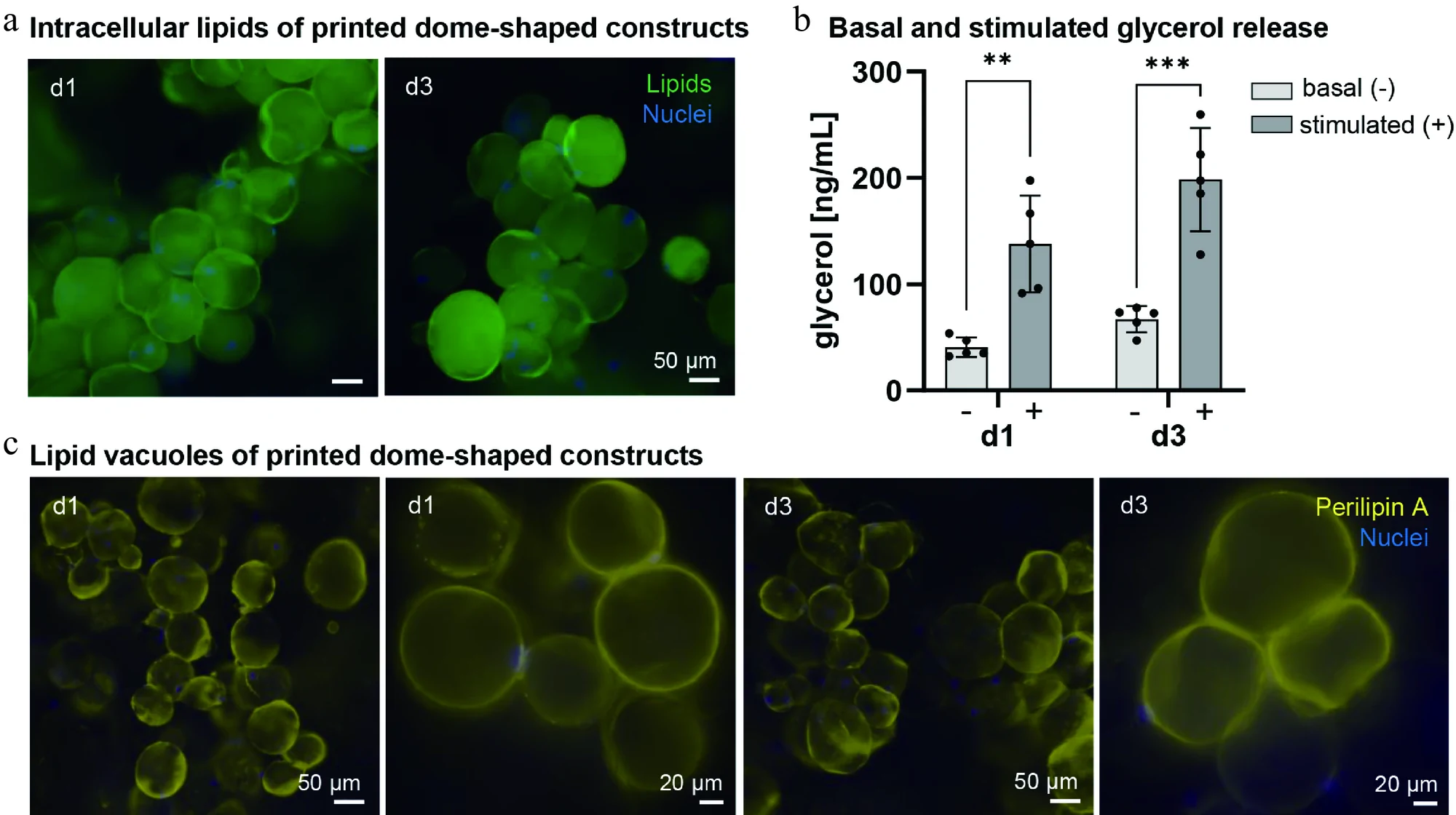
Morphology and function of mature adipocytes in the printed dome-shaped construct. (a) Intracellular lipid (BODIPY) staining of printed dome-shaped constructs on days 1 and 3, lipids in green, cell nuclei in blue. Scale bar: 50 µm. (b) Basal (−) and isoproterenol-stimulated (10 µM) (+) glycerol release of dome-shaped AC constructs on days 1 and 3 post-printing. (c) Lipid vacuole (perilipin A) staining of printed dome-shaped constructs with two different magnifications (20x and 40x), lipid vacuoles in yellow, nuclei in blue. Scale bars: 50 µm and 20 µm. Mean values are shown as mean ± standard deviation, with individual values as dots from five independent experiments with five different biological donors. ** *p* ≤ 0.01, *** *p* ≤ 0.001.

Besides measuring viability to evaluate the suitability of the established printing protocol for AC tissue engineering, preserving the unilocular AC morphology and functionality are essential criterion. Intracellular lipid staining showed an aligned distribution of homogeneously lipid-filled cells over 3 d (Fig. 5a). The functionality of the printed AC constructs was evaluated by comparing basal and isoproterenol-stimulated (10 µM) glycerol release on days 1 and 3 post-printing (Fig. 5b). The basal glycerol release on day 1 was 40.60 ± 11.61 ng/mL, with a slight increase on day 3 with 65.86 ± 14.95 ng/mL. The isoproterenol-stimulated glycerol release was significantly higher than the unstimulated release (d1: 137.88 ± 45.13 ng/mL, d3: 200.54 ± 47.40 ng/mL), which indicates an intact and reactive metabolic function of the printed ACs. The lipid vacuole integrity was evaluated by perilipin A staining. On both evaluation days (days 1 and 3), a uniform coating of the lipid vacuoles was visible, indicating intact vacuole integrity (Fig. 5c). Printed and recovered AC constructs met all criteria for intact biological morphology and function, demonstrating that the here-established printing protocol is useful for constructing adipose cell models using ACs.

## Discussion

This study demonstrates that SB extrusion bioprinting combined with tunable GG-based bioinks provides a robust strategy for fabricating geometrically defined constructs with viable, functional ACs. These cells are among the most difficult to process in biofabrication because of their high buoyancy, large lipid droplets, and sensitivity to shear stress. However, using ACs for *in vitro* models and soft tissue reconstruction offers several benefits over stem cells, including immediate availability after isolation and a fully differentiated, metabolically active phenotype. By characterizing both the support bath and bioink properties, and by linking material behavior to printing performance and biological outcomes, this work presents a reproducible workflow for printing lipid-laden cells while preserving construct accuracy and cellular integrity.

### Rheological properties of agarose support bath and GG-based bioink enable a low-shear printing environment

A goal of this work was to identify a rheological window in which ACs can be gently extruded without too much mechanical damage. The GG solution with 20% PBS^+^ showed higher viscosity at low shear rates but exhibited a sharper decrease in viscosity as shear rate increased, resulting in lower viscosity at higher shear rates compared to the 10% PBS^+^ formulation. These differences may be due to variations in ionic interactions and network structure caused by PBS^+^ concentrations, which influence how the material responds to applied shear^[18,19]^. The shear-thinning behavior of the GG solution allows it to flow under low extrusion pressures, consistent with previous studies showing that shear-thinning hydrogels enable gentle cell encapsulation during printing^[11]^. The support bath provided a yield-stress, self-healing environment, consistent with the reported behavior of suspension baths used in embedded bioprinting^[20,21]^. The combination of these rheological profiles created a low-shear, high-fidelity printing environment that addressed the primary limitations of printing mature adipocytes. These findings align with other studies using SBs, which indicate that yield-stress SBs such as gelatin microparticles, Carbopol, or Pluronic^[21]^ facilitate embedded printing of soft and fragile materials by providing reversible, on-demand mechanical confinement. However, this study is among the few to apply these principles specifically to the unique constraints of AC bioprinting.

### Support-bath bioprinting improves construct geometry and fidelity

A central outcome of this study is the improvement in geometric accuracy when printing in an SB compared to free extrusion. Printed grid structures without the SB showed filament spreading, irregular pore shapes, and poor filament stacking due to the inability of the low-viscosity bioink to resist gravity and surface tension forces. In contrast, SB printing yielded filaments with significantly reduced dimensional deviation, improved pore uniformity, and greater interlayer alignment. These improvements in fidelity directly reflect the material-centric optimization of printing parameters. The SB prevented filament drift, which is consistent with prior reports demonstrating improved fidelity when printing soft or buoyant materials in embedded environments^[22,23]^. Furthermore, the successful biofabrication of dome-shaped constructs demonstrates the method's ability to maintain 3D geometry, a crucial feature for mimicking physiologically relevant adipose microarchitectures^[24,25]^. The reduced definition of strands and pores in the 3D constructs compared to the planar grid structures reflects a design emphasis on cohesive shape formation rather than a limitation of the support bath approach. Similar trends have been reported for embedding systems, where excellent planar fidelity transitions toward increased filament integration in taller, densely packed constructs due to cumulative deposition and confinement effects within the support medium^[22,26,27]^. While this enhances mechanical stability, it may reduce macroporosity at higher infill densities, as also observed in FRESH-based strategies^[28]^. Prior work shows that macroporosity can be reintroduced through parameter tuning or the use of sacrificial elements, highlighting clear potential for optimization. This work adds to the growing evidence that geometric fidelity in bioprinting must be viewed as a coupled response between bioink rheology, SB mechanics, and printing conditions. The analysis of these parameters provides a foundation for reproducible fabrication of adipose constructs and practical guidelines for researchers working with extremely soft or buoyant cellular materials.

### Gentle extrusion preserves adipocyte viability and retains unilocular morphology and metabolic function

LDH levels, which decreased from day 1 to day 3, could reflect minimal membrane damage or also a reduced cell number over the first 72 h of culture after printing. However, live/dead staining on day 3 shows a predominance of viable cells with no indication of increased cell debris, arguing against substantial cell loss. Together with the previously described transient increase in LDH following bioprinting-induced membrane stress, which decreases as cells reseal and adapt^[29]^, this supports interpreting the LDH decline as an indicator of post-printing recovery. This is a significant achievement given that mature adipocytes are particularly prone to rupture under mechanical stress. The low extrusion pressures and shear-minimizing SB environment appear critical to maintaining cell integrity. Compared with conventional extrusion, in which lipid droplets often collapse or cells delipidate post-printing, the SB approach sustains both cell viability and intracellular lipid morphology. These findings align with recent reports demonstrating that embedded printing techniques reduce hydrodynamic stress^[10]^, yet this study provides one of the few demonstrations of this principle in ACs. Immunofluorescence staining for BODIPY and perilipin A showed that printed ACs retained the characteristic round morphology and unilocular lipid droplet organization typical of *in vivo* adipocytes. Perilipin A, a marker of lipid droplet membrane integrity^[30]^, remained strongly expressed across printed constructs, suggesting that neither extrusion nor embedding disrupted lipid packaging or membrane-associated proteins. Glycerol release assays further confirmed active metabolic function^[31]^, indicating that adipocytes preserved basal and reactive lipolytic activity after printing. Maintaining such metabolic competence is crucial for applications in metabolic disease modeling, drug testing, and adipose physiology research^[32−34]^. Together, these results demonstrate that SB printing preserves both structural and functional hallmarks of ACs, in contrast to traditional techniques that often induce dedifferentiation or mechanical injury^[35,36]^.

### Implications for adipose tissue engineering and future perspectives

This study focused on short-term outcomes that can be immediately used for biofabrication of adipose cell models. Extending the culture period beyond 3 d could enable more in-depth analysis of long-term effects on lipid metabolism, cell-matrix remodeling, and construct stability. The model's potential for long-term culture was briefly investigated in this study by assessing its viability at day 8. The absence of perfusion and vascularization limits this model to smaller geometries, which could be improved by developing the technique with additional material-cell combinations. Therefore, future work should explore co-printing with fibroblasts, endothelial cells, and/or immune components to mimic the complex cellular interactions of native adipose tissue. Incorporating vascular components into multicellular approaches to achieve greater biological fidelity is essential. Additionally, technical improvements of the SB formulation, such as enhanced optical clarity, may facilitate better multi-material integration. Nonetheless, this study provides an accessible, material-based protocol for reproducible 3D adipose tissue models capable of delivering custom geometries for preclinical testing in disease research and potential for clinical applications in reconstructive surgery and cosmetic testing.

## Conclusions

This study demonstrates that support-bath bioprinting with a gellan gum-based bioink enables the precise fabrication of 3D constructs containing mature adipocytes while preserving their viability, morphology, and metabolic function. Compared with free extrusion, support-bath printing improved structural fidelity and print quality, providing a gentle environment for these fragile, lipid-laden cells.

Overall, the developed workflow offers a reproducible and accessible platform for generating physiologically relevant adipose cell models. The resulting constructs provide a promising preclinical test system for investigating adipose tissue biology, modeling metabolic dysfunction, and evaluating therapeutic compounds in a controlled 3D environment.

## SUPPLEMENTARY DATA

Supplementary data to this article can be found online.

## Data Availability

The data that support the findings of this study are available from the corresponding author upon reasonable request.
