# Supplementary material for: Engineering 3D adipose cell models by support bath bioprinting of mature adipocytes in gellan gum hydrogels
Source: Eng Life Sci. 2026 Aug 10;26(8):e006. doi: 10.48130/els-0026-0006 (PMC13599641; doi:10.48130/els-0026-0006)
Supplement: Supplementary file 1 [file els-2026-26-8-0006-S1.zip › 10.48130_els-0026-0006-Suppl-FigureS3.pdf]

A Viability of dome-shaped constructs d8 – single channels

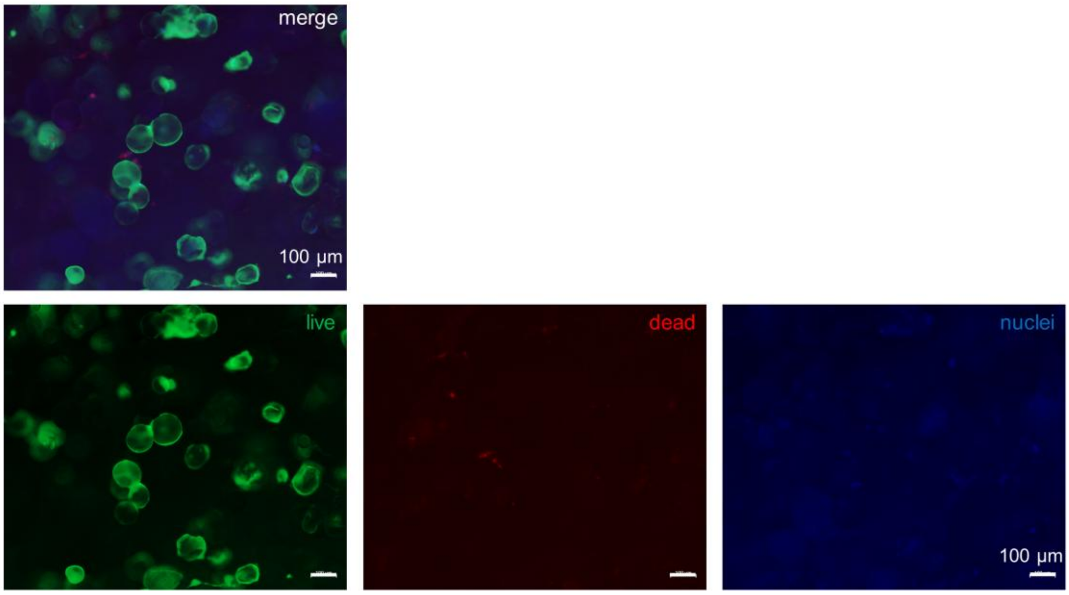

Figure S3: Viability of mature adipocytes in the printed dome-shaped constructs after 8 days of culture.

A| Live/dead staining of printed dome-shaped constructs on day 8 post-printing, scale bar: 100 µm. Single channels of live cells (green), dead cells (red) and nuclei (blue) are displayed. The pictures are representative of three different experiments with three different biological donors.
