# Supplementary material for: Engineering 3D adipose cell models by support bath bioprinting of mature adipocytes in gellan gum hydrogels
Source: Eng Life Sci. 2026 Aug 10;26(8):e006. doi: 10.48130/els-0026-0006 (PMC13599641; doi:10.48130/els-0026-0006)
Supplement: Supplementary file 1 [file els-2026-26-8-0006-S1.zip › 10.48130_els-0026-0006-Suppl-TableS1.pdf]

Table S1: Donor demographics.

Donor	Sex	Age	Source of biopsy	BMI at surgery
Only used for grid structure				
1	Female	52	Abdomen	24.5
Used for grid structure and dome-shaped constructs				
2	Female	56	Upper arm	27.1
3	Female	37	Upper arm	30.1
4	Female	42	Upper arm	25.6
5	Female	44	Thigh	22.7
6	Female	40	Thigh	29.07
