# Supplementary material for: Engineering 3D adipose cell models by support bath bioprinting of mature adipocytes in gellan gum hydrogels
Source: Eng Life Sci. 2026 Aug 10;26(8):e006. doi: 10.48130/els-0026-0006 (PMC13599641; doi:10.48130/els-0026-0006)
Supplement: Supplementary file 1 [file els-2026-26-8-0006-S1.zip › 10.48130_els-0026-0006-Suppl-FigureS1.pdf]

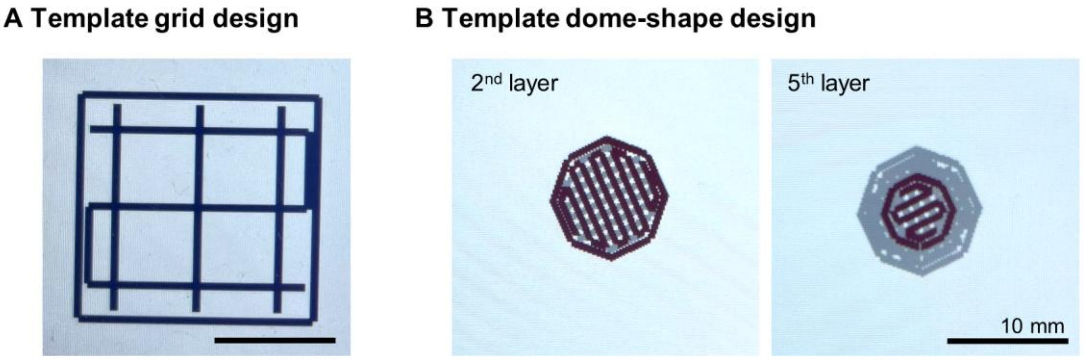

Figure S1: Templates of grid (A) and dome-shaped design (B) on the BioX printer, scale bar: 10 mm.
